# Comparison of self-refraction using a simple device, USee, with manifest refraction in adults

**DOI:** 10.1371/journal.pone.0192055

**Published:** 2018-02-01

**Authors:** Anvesh Annadanam, Varshini Varadaraj, Lucy I. Mudie, Alice Liu, William G. Plum, J. Kevin White, Megan E. Collins, David S. Friedman

**Affiliations:** 1 Dana Center for Preventive Ophthalmology, Wilmer Eye Institute, Johns Hopkins School of Medicine, Baltimore, Maryland, United States of America; 2 Global Vision 2020, Easton, Maryland, United States of America; The University of Melbourne, AUSTRALIA

## Abstract

**Background:**

The USee device is a new self-refraction tool that allows users to determine their own refractive error. We evaluated the ease of use of USee in adults, and compared the refractive error correction achieved with USee to clinical manifest refraction.

**Methods:**

Sixty adults with uncorrected visual acuity <20/30 and spherical equivalent between –6.00 and +6.00 diopters completed manifest refraction and self-refraction.

**Results:**

Subjects had a mean (±SD) age of 53.1 (±18.6) years, and 27 (45.0%) were male. Mean (±SD) spherical equivalent measured by manifest refraction and self-refraction were –0.90 D (±2.53) and –1.22 diopters (±2.42), respectively (p = 0.001). The proportion of subjects correctable to ≥20/30 in the better eye was higher for manifest refraction (96.7%) than self-refraction (83.3%, p = 0.005). Failure to achieve visual acuity ≥20/30 with self-refraction in right eyes was associated with increasing age (per year, OR: 1.05; 95% CI: 1.00–1.10) and higher cylindrical power (per diopter, OR: 7.26; 95% CI: 1.88–28.1). Subjectively, 95% of participants thought USee was easy to use, 85% thought self-refraction correction was better than being uncorrected, 57% thought vision with self-refraction correction was similar to their current corrective lenses, and 53% rated their vision as “very good” or “excellent” with self-refraction.

**Conclusion:**

Self-refraction provides acceptable refractive error correction in the majority of adults. Programs targeting resource-poor settings could potentially use USee to provide easy on-site refractive error correction.

## Introduction

Uncorrected refractive error (RE) is the most common cause of preventable visual impairment (VI) globally [1]. The measurement of refractive error typically requires an eyecare specialist, and this need for technical expertise is a potential limiting factor in the provision of eyeglasses in resource-poor areas of the world as many communities do not have consistent access to high quality refractive services. A study performed in rural Southern-India found that >65% of a population with high rates of RE correctable with glasses were not wearing them due to inadequate access [2]. As such, the World Health Organization and the International Agency for the Prevention of Blindness have made the treatment of RE a priority in their VISION 2020 global initiative [3]. In 2011, however, many countries in sub-Saharan Africa were not on target to achieve goals of 20 refractionists per million population due to a smaller and more unevenly distributed eye health workforce [4].

Good vision is necessary for success in education and work, and maintaining health. The annual loss in global gross domestic product due to distance VI caused by uncorrected RE was estimated at $202 billion in 2007, while the cost for training personnel and purchasing equipment to correct these errors would be only $20–28 billion [5]. Despite the enormous potential benefit of correcting RE, the lack of trained professionals remains an important obstacle to vision care in low resource settings [4, 6]. In the United States (US), there is approximately one eyecare professional for every 5500 people, while in parts of Africa and rural India, the ratio is closer to one refractionist per one million people and 219,000 people, respectively [7–9].

While clinical manifest refraction (MR) is ideal for RE correction, newer technologies allowing for self-refraction (SR) may be a cost-effective substitute that requires fewer resources [10]. USee is a device that is worn like a pair of glasses and has a graded lens bar that the user can move up or down to adjust the spherical equivalent (SE) refractive power in each eye. USee allows a user to self-refract and receive a pair of plastic eyeglasses on site with pop-in best-sphere lenses, an improvement over ready-made spectacles used in some programs. Although USee can only provide SE correction, prior studies have shown that ready-made eyeglasses of this type were still well tolerated and highly valued [3, 11–15].

In this first study with the USee, we report on the accuracy and ease of use in measuring RE among adults in optometry clinics in the US. Although not the ultimate intended patient population, this study allowed for development and validation of a protocol for using the USee device, which will be applied in future studies in its target population.

## Materials and methods

The Johns Hopkins Hospital Institutional Review Board approved this study. It was HIPAA compliant and followed the tenets of the Declaration of Helsinki. Informed written consent was obtained prior to all study procedures.

### Participants and setting

Participants comprised English-speaking patients of optometrists of the Wilmer Eye Institute, recruited in June-July 2016. Patients were excluded if they had (1) undergone eye surgery in the last 30 days, (2) uncorrected vision ≥20/30 in either eye, (3) best corrected vision <20/400 in either eye, (4) coexisting ocular pathology causing VI (cataract, macular degeneration etc.), and (5) known SE RE > +6.00 diopters (D) or < –6.00 D, due to device limitations. All testing conditions, including lighting, distance between subject and eye chart, and visual acuity measurement protocol, were identical for each subject. On average, measurement of visual acuity and refractive errors were completed within 15 minutes. The study protocol was completed in the order described below.

### Visual acuity measurement

Measurement of distance visual acuity (VA) was first tested with and without existing corrective lenses (if worn) using computerized ETDRS charts (Innova Systems, Burr Ridge, IL) with luminance in the range of 0–4.3 candelas/m^2^, and placed 3 m in front of the subject. In the interest of brevity, a modified ETDRS protocol was used to measure VA [16]. The same chart was used for both MR and SR VA measurements. Starting at the 20/40 line on the chart, monocular and binocular testing proceeded sequentially to the lowest line at which ≥50% of letters were read correctly. If patients were unable to read the 20/40 line, line size was increased sequentially until patients could read ≥50% of letters on a given line.

### Self-refraction

**Fig 1:**
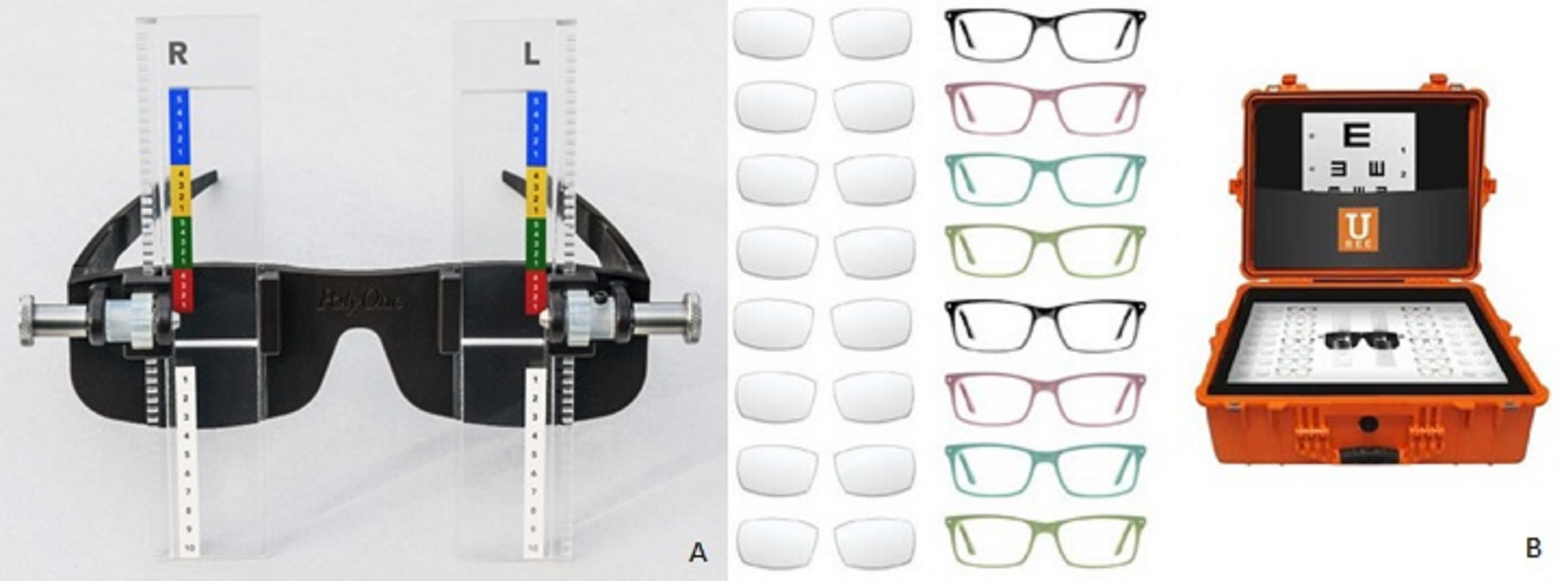
USee device and kit. (A) USee device with dials that move lens bar to appropriate refractive power, (B) USee EyeOpener kit including USee device, plastic frames, pop-in lenses, and visual acuity chart.

The SR device (USee, Global Vision 2020, Easton, MD) uses a single, durable, adjustable progressive lens for each eye placed directly in front of a 3mm rectangular opening slit in an eyeglass frame. The refraction bar is toggled up and down using a dial on each side of the frame. Each adjustment level of the dial on the frame used to change optical power is marked by an easy to use color and number combination corresponding to a specific SE power (Fig 1). The optical power of the lens is determined by the curvature of the lens surface. A refractive power SE ranging from –6.00 to +6.00 D is achievable, but cylindrical correction is not possible. In the myopic direction, increments of 0.25 D are available from –0.5 to –3.5 D and 0.5 D increments from –3.5 to –6.0 D. In the hyperopic direction, 0.5 D increments are available from +1.0 to +6.0 D.

Trained, non-clinical research personnel observed as the subjects completed the SR protocol. The refraction bar was set to the most positive setting (+6.00 D) and subjects were instructed to slowly turn the dial toward themselves (reducing plus power) until letters on the chart appeared clearest, and to make fine adjustments as necessary. VA was measured using the above protocol, while the subject looked through USee using the self-selected optical power, both monocularly and binocularly. This was considered the preliminary USee result.

In an attempt to uncover any “overminus” bias inherent to the device, the duochrome test [17] was used. A line of letters was shown on the screen, split by a left-sided red background and a right-sided green background. If letters on the green or red side appeared clearer, 0.25 D was added or subtracted to the preliminary USee result, respectively, until both sides were equally clear to the user. This was determined the final USee result. Plastic lenses matching these RE measurements were snapped into a plastic frame (Vision Spring, New York, NY) and VA was re-measured monocularly and binocularly. This was considered the final SR VA. The plastic frames had an inter-pupillary distance of 64 mm. Appropriately powered plastic pop-in lenses were unavailable for at least one eye of nine subjects. For those eyes, SE and VA determined by USee post-duochrome were used as the final measurements.

Finally, inter-pupillary distance was measured using a digital device (Besmic, Zhejiang, China).

### Clinical refraction

MR was performed using a phoropter by a licensed optometrist masked to USee results. VA was measured as described above. The experimental setup was replicated in each optometrist’s office for the measurement of MR.

### Statistical analysis

In order to detect a minimum difference in SE between SR and MR of 0.5 D, with a two-sided significance of 0.05 and a power of 0.8, we required a sample size of 62 subjects.

The primary outcome variable was best corrected VA (BCVA) coded as a binary variable, ≥20/30 vs. <20/30. In general, 20/40 is used internationally as the visual acuity cutoff for legal driving without restriction, so 20/30 was chosen as an acceptable threshold for good vision in our study [18]. Wilcoxon rank test was used to assess differences in the proportion of participants achieving a BCVA of ≥20/30 in the better eye for MR and SR. For further regression analyses, only right eye data was uniformly analyzed as right and left eye data are correlated. Univariable and multivariable logistic regressions were used to determine the association of age and cylindrical power as predictors of failure to achieve VA of ≥20/30 with SR. SE differences between SR and MR were illustrated using modified Bland-Altman graphs [19]. Regression analysis was also conducted to analyze the association of age and cylindrical power with having SR measurements differ by ≥1.00 D in either direction from MR SE. Univariable logistic regression was used to determine the association of differences in inter-pupillary distance with failure to achieve ≥20/30 VA and differences between MR and SE of ≥1.00 D. Logistic regression was also used to test the association between age and the likelihood of having a cylindrical error. The Spearman correlation test was performed to analyze the monotonic relationship between MR SE and SR.

Participants provided subjective feedback in the form of a 5-point Likert scale regarding ease of use of USee and satisfaction with their vision achieved with USee correction [20]. The proportion of patients indicating an 80% or higher response to each question was analyzed.

All analyses were performed using STATA 14 (StataCorp LP, College Station, TX).

## Results

Sixty-seven adult participants consented for the study, of which 60 (89.6%) were deemed eligible based on set criteria. Subjects comprised myopes and hyperopes with a mean (±SD) age of 53.1 (±18.6) years and 27 (45.0%) were male (Table 1). More than half (61.7%) presented to the study visit with prescription corrective lenses.

**Table 1:** Distribution of age, gender, handedness, ethnicity, and race among study participants (N = 60).

**Age** (years)	n (%)
18–39	17 (28%)
40–64	25 (42%)
65+	18 (30%)
**Gender:** (male)	27 (45%)
**Handedness**	
Right	54 (90%)
Left	5 (8%)
Ambidextrous	1 (2%)
**Ethnicity**	
Non-Hispanic	56 (93%)
Hispanic	2 (3%)
Declined to Answer	2 (3%)
**Race**	
White	36 (60%)
Black	18 (30%)
Asian	4 (7%)
Declined to Answer	2 (3%)
**Total**	60 (100%)

For the better and worse seeing eyes, median uncorrected VA was logMAR (Snellen) 0.40 (20/50) and 0.60 (20/80), respectively (Table 2, S1 Fig). Both SR and MR improved uncorrected VA by a median 0.40 logMAR units in the better eye, which is an improvement of four lines on the chart. The proportion of subjects correctable to ≥20/30 in the better-seeing eye for SR and MR was 83.3% and 96.7%, respectively (p = 0.005). The mean (±SD, range) SE measured by SR and MR for right eyes was –1.22 D (±2.42, –6.00 to 3.00) and –0.90 D (±2.53, –5.75 to 3.875), respectively (p = 0.001, S2 Fig). The mean (±SD, range) SE measured by SR and MR for left eyes was –1.37 D (±2.62, –6.00 to 4.00) and –1.15 D (±2.74, –6.00 to 5.00), respectively (p = 0.066). The mean cylinder measured by MR was 0.68 D and 0.74 D for right and left eyes, respectively (S3 Fig). Older age was associated with a higher likelihood of having cylindrical error (p = 0.005).

**Table 2:** Distribution of visual acuity before and after refraction.

	Uncorrected VA		USee BCVA		Clinical BCVA	
Snellen VA (logMAR)	Better eye	Worse eye	Better eye	Worse eye	Better eye	Worse eye
20/20 (0)			41	26	54	46
20/25 (0.10)			9	12	4	8
20/30 (0.18)			6	9	2	4
20/40 (0.30)	16	7	4	11		2
20/50 (0.40)	15	8		1		
20/60 (0.48)	9	10		1		
20/70 (0.54)	1	1				
20/80 (0.60)	1	5				
20/100 (0.70)	3	5				
20/125 (0.80)	1	1				
20/150 (0.88)	2	3				
20/200 (1.00)	6	13				
20/250 (1.10)	1	1				
20/300 (1.18)	1					
20/400 (1.30)	1	2				
20/500 (1.40)	1					
20/600 (1.48)		1				
Count Fingers (2.00)	2	3				
Median (IQR) VA	0.40 (0.45)	0.60 (0.56)	0.00 (0.10)	0.10 (0.18)	0.00 (0)	0.00 (0)
Mean (SD) VA	0.60 (0.39)	0.75 (0.41)	0.05 (0.09)	0.12 (0.13)	0.01 (0.04)	0.04 (0.07)

VA = Visual acuity; BCVA = Best corrected visual acuity; IQR = Inter-quartile range; SD = standard deviation

The mean difference between SR and MR in participants less than 40 years old was 0.10 D and 0.06 D in right and left eyes, respectively. In univariable analysis, an age of ≥66 years carried higher odds of achieving vision worse than 20/30 in the right eye with SR compared to an age of <40 years (OR: 6.00, 95% CI: 1.05 to 34.3, p = 0.044). A cylindrical power of >1.00 D was associated with vision worse than 20/30 with SR compared to that of <0.50 D in both univariable (OR: 21.3, 95% CI: 1.91 to 236, p = 0.013) and multivariable (OR: 13.3, 95% CI: 1.08 to 165, p = 0.044, Table 3) analyses. A difference of ≥ ±1.00 D between SR and MR SE was present in 18.3% and 26.7% of right and left eyes, respectively, but this was not significantly associated with age or cylindrical power (p>0.05).

**Table 3:** Predictors of failure to achieve better than or equal to 20/30 visual acuity in the right eye.

Variable	Univariable OR (95% CI)	p value	Multivariable OR^a^ (95% CI)	p value
Age (years)				
< 40	Reference	-	Reference	-
40–65	1.88 (0.32–11.0)	0.487	1.37 (0.21–8.89)	0.739
66+	6.00 (1.05–34.3)	**0.044**^**b**^	2.99 (0.46–19.6)	0.254
Cylindrical power (D)				
0–0.25	Reference	-	Reference	-
0.5–1.0	6.38 (0.74–55.1)	0.092	4.55 (0.49–42.4)	0.184
1.25+	21.3 (1.91–236)	**0.013**^**b**^	13.3 (1.08–165)	**0.044**^**b**^

OR = odds ratio

^a^Covariates included age and cylindrical power

^b^Indicates p ≤ 0.05

**Fig 2:**
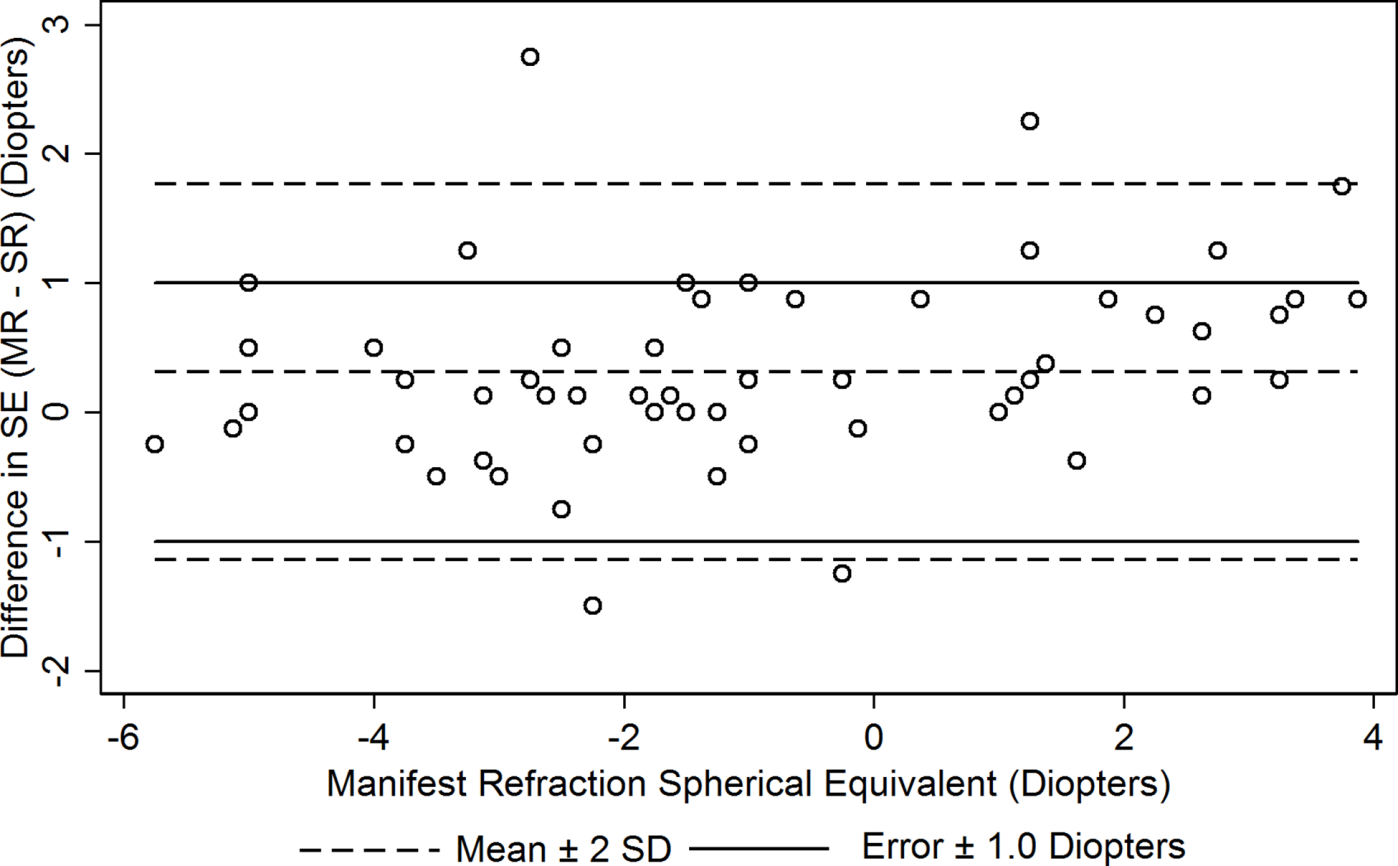
Modified Bland Altman (Difference in SE (MR SE minus SR) vs. MR SE) for right eye. Abbreviations: SE = spherical equivalent, MR = manifest refraction, SR = self-refraction.

Fig 2 shows a modified Bland Altman graph comparing SR to MR. The mean (±SD) difference for right eyes was 0.31 (±0.73) D (Wilcoxon signed rank test, p = 0.001) and 0.23 (±0.88) D for left eyes (p = 0.07).

On the duochrome test used to identify any overminus refractions by SR, 63.3% of subjects had no difference in their refraction after the test for the right eye, and only 5% had a difference of >0.5 D in either direction. For the left eye, 73.3% had no difference and 5% had a difference of >0.5 D in either direction.

Subjectively, 95% of subjects thought USee was easy to use, 85% thought the SR correction was better than being uncorrected, 57% thought the SR correction was as good as their current corrective lenses, and 53% rated their vision as “very good” or “excellent” with SR correction (Table 4). The mean right eye cylinder in those that rated ≤3 in question 3 was 0.71 D, whereas it was 0.65 D in those that scored a 4 or 5 (p = 0.568). Subjective comments by those who gave lower ratings included that their current corrective lenses were sharper and crisper than the pop-in lenses.

**Table 4:** Subjective feedback on USee.

Question^a^	Scored 1 or 2 [n (%)]	Scored 3 [n (%)]	Scored 4 or 5 [n (%)]
1. The USee was easy to use	2 (3.3)	1 (1.7)	57 (95)
2. Compared to no correction, the pop-in glasses made my vision better	4 (6.7)	5 (8.3)	51 (85)
3. The pop-in glasses work as well as my current correction	15 (25)	9 (15)	34 (56.7)
4. How would you rate your vision with the pop-in glasses?	11 (18.3)	17 (28.3)	32 (53.3)

^a^For questions one to three, 1 = strongly disagree, 2 = disagree, 3 = neutral, 4 = agree, 5 = strongly agree. For question four, 1 = poor, 2 = fair, 3 = good, 4 = very good, 5 = excellent

Finally, differences in subjects’ inter-pupillary distance were not significantly associated with achieving vision <20/30 (p = 0.352) or having a difference of ≥ ±1.00 D between SR and MR (p = 0.285).

## Discussion

This study compared VA and RE measurements with a new SR device to those obtained by gold-standard MR. More than 80% of the study population was able to obtain a VA of ≥20/30 in the better seeing eye with USee, confirmed using pop-in lenses. Better visual outcomes with MR compared to SR are expected, as MR corrects for astigmatism, allowing for more precise RE assessment. Furthermore, a trained refractionist can more accurately adjust refractive power, thus reducing overminusing. As expected, higher cylindrical error was associated with failure to achieve optimal vision in this study as has been the case in other SR studies [3, 11]. Poorer SR visual outcomes in older subjects may be due to a steeper learning curve for using USee. In addition, older subjects were more likely to have a cylindrical error as compared to younger subjects.

Previous studies have used other SR technologies such as the AdSpecs (Centre for Vision in the Developing World) and FocusSpecs (FocusSpecs LLC, Durham, NC). USee users achieved vision levels similar to those reported with previous approaches, including one study in Ghana (FocusSpecs) [21], and two AdSpecs studies from China [3, 11]. Currently, we are unaware of any other portable, mechanical SR technologies that are able to provide refractive error correction beyond the –6 to +6 D range offered by USee.

USee resulted in overminusing by a mean of 0.31 D for SR compared to MR, likely due to accommodation in younger subjects and reasons previously mentioned for older subjects. SR accuracy may also be dependent on learning, as demonstrated by du Toit et al, who studied repeated RE measurements using the Focometer and found that using three trials resulted in greater accuracy than a single trial [22], Although our participants were only instructed once on how to use USee, the majority were able to correct themselves to within 1.00 D of MR, comparable to other studies [3, 11, 21]. We expect that repeated uses of USee for an individual would result in a closer approximation to MR. Overall, while the USee was well received, only 57% of subjects thought the trial frames with SR correction worked as well as their current prescriptions. This is likely in part attributable to better astigmatic correction and the perception of more subjective clarity offered by their existing corrective lenses. Although our overall test population’s prevalence of astigmatism, defined as ≥1.0 D of cylinder in either eye, was higher than in the general United States population (48.3% vs. 36.2%) [23], the mean cylinder was not significantly different in those that gave the pop-in lenses a higher versus lower rating. As seen in Fig 2, there seems to be a suggestion toward greater myopic overcorrection in hyperopes versus myopes for SR. However, we note that this is still within the clinically acceptable error of ±1.00 D from MR. This phenomenon was also noted in the study by He, et al [3].

We utilized the duochrome test to identify any inherent overminusing effect of USee, however, few subjects had a clinically significant change in RE on the basis of duochrome testing. This test is not intended to be utilized during field usage of USee. In order to address this issue further, we evaluated 12 additional subjects (data not presented) and measured VA with two separate trial frames: one that matched SR, and one that was 0.5 D more positive than SR in both eyes as a universal overminus correction. If VA was the same or better with the second frame, it was used as the final USee refraction, as done in 4 of the 12 patients. Outcomes of BCVA and SE difference between MR and SR were similar to that of the original cohort studied. Thus, a systemic compensation in order to avoid overminusing did not help decrease the degree of myopic bias in USee refraction.

The study population was tested in a US clinic setting with many having good access to eyecare and a lifetime of refractive correction based solely on MR. This does not necessarily reflect the demographic that might benefit from USee in the future. However, the RE distribution represented in our study mirrored those found in other studies of prevalence of RE in adults in rural areas around the world [24–26]. Subjects’ MR was measured by several different optometrists. However, all optometrists were board certified and were in practice for at least 5 years, and the experimental setup was identical in each clinic room used. Previous literature has shown that inter-examiner determination of MR is within acceptable limits [27], and our study did not show an association between the extent of error between USee and MR and the optometrist performing refraction. We did not assess the test-retest reliability of the USee measurement or acceptability of the trial frames for long-term use. Such studies are planned for future field trials of the device. Furthermore, SR technology does not claim to replace the full ophthalmology exam done to detect other ocular pathologies. It is possible that vision loss due to eye diseases other than uncorrected RE may occur.

Current estimates place the cost of the USee system, including the USee device, plastic frames, pop-in lenses, and VA chart at USD 5. This VA chart is clinically equivalent to that used in this study’s measurement of MR and is not predicted to have variability in measuring SR. Plastic frames of varying inter-pupillary diameters will be available to meet the needs of a wide range of individuals. USee does not require electricity or battery power, and is more portable and cost effective than other available SR tools as well as tabletop or handheld autorefractors. USee could be used at vision screening sites in low-resource communities where trained vision professionals are scarce. Schoolteachers and community leaders have previously helped conduct vision screenings in order to provide glasses to young children in low resource areas and it is possible that they could be trained to use USee to measure RE and distribute glasses [3, 11, 21, 28].

In summary, the USee is easy to use, provides significantly improved VA compared to uncorrected vision, and results in vision that is 20/30 or better in over 80% of users. This system, which allows for instant distribution of glasses and immediate feedback on vision quality, could help reduce the burden of uncorrected RE in low-resource communities.

## Supporting information

S1 Fig Distribution of visual acuity before and after refraction. Abbreviations: OD = right eye, OS = left eye.
(TIF)

S2 Fig Distribution of refractive error in right and left eyes. Abbreviations: MR = manifest refraction, SE = spherical equivalent, OD = right eye, OS = left eye.
(TIF)

S3 Fig Distribution of cylindrical error in right and left eyes. Abbreviations: OD = right eye, OS = left eye.
(TIF)
