# Supplementary figures and images for: Comparison of self-refraction using a simple device, USee, with manifest refraction in adults
Source: PLoS One. 2018 Feb 1;13(2):e0192055. doi: 10.1371/journal.pone.0192055 (PMC5794143; doi:10.1371/journal.pone.0192055)

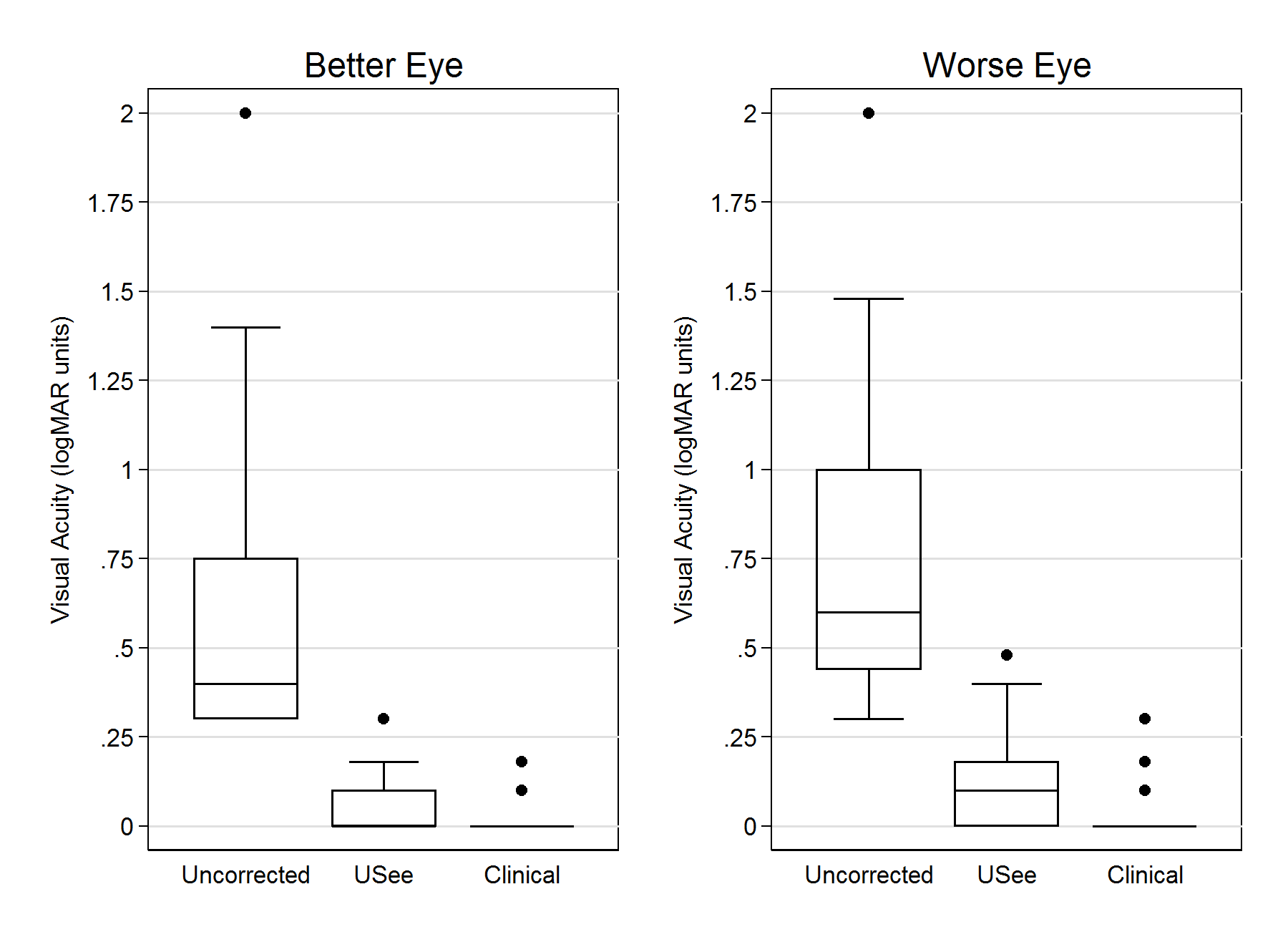

Supplement: S1 Fig — Abbreviations: OD = right eye, OS = left eye. (TIF) [file pone.0192055.s001.tif]

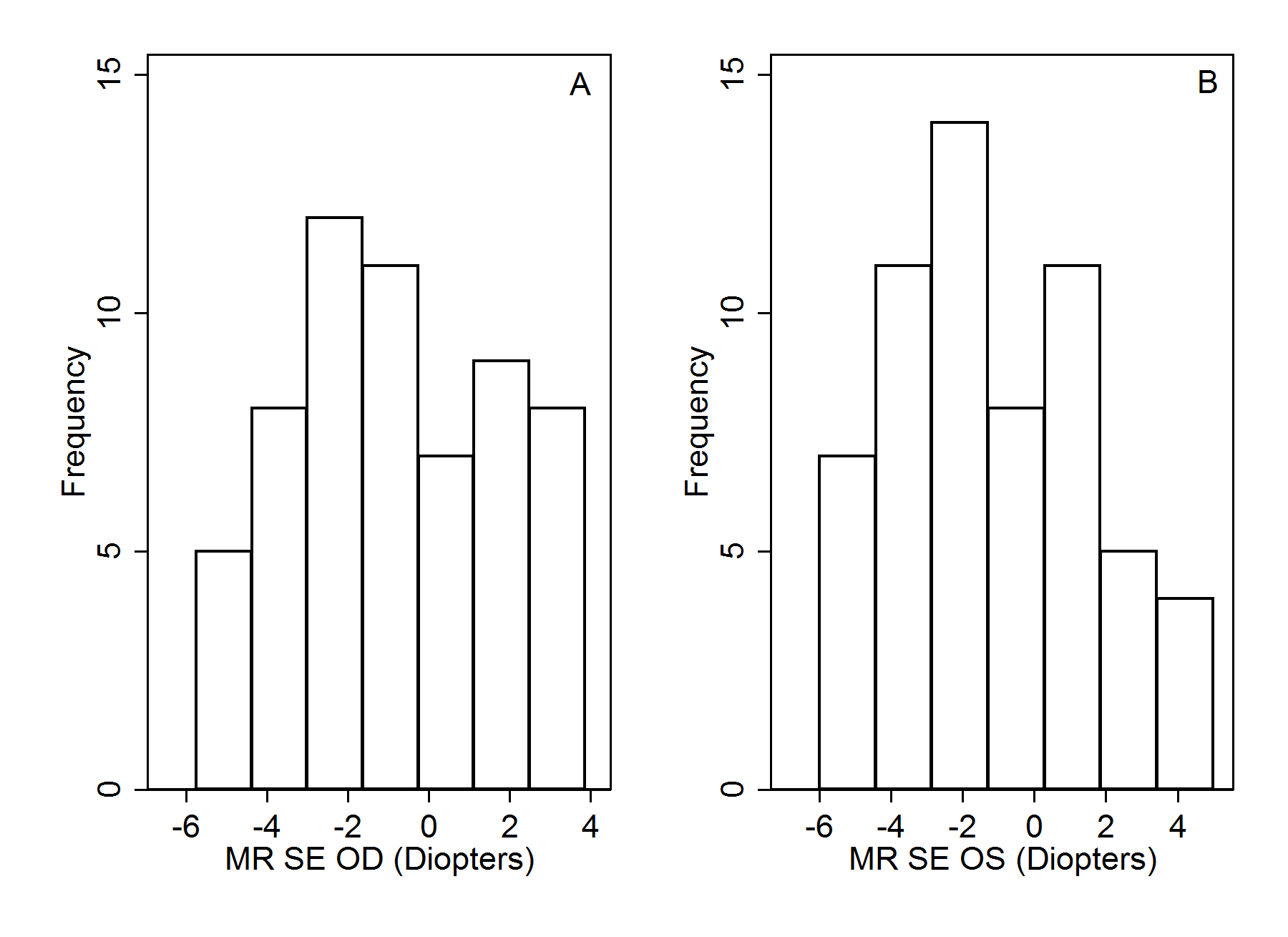

Supplement: S2 Fig — Abbreviations: MR = manifest refraction, SE = spherical equivalent, OD = right eye, OS = left eye. (TIF) [file pone.0192055.s002.tif]

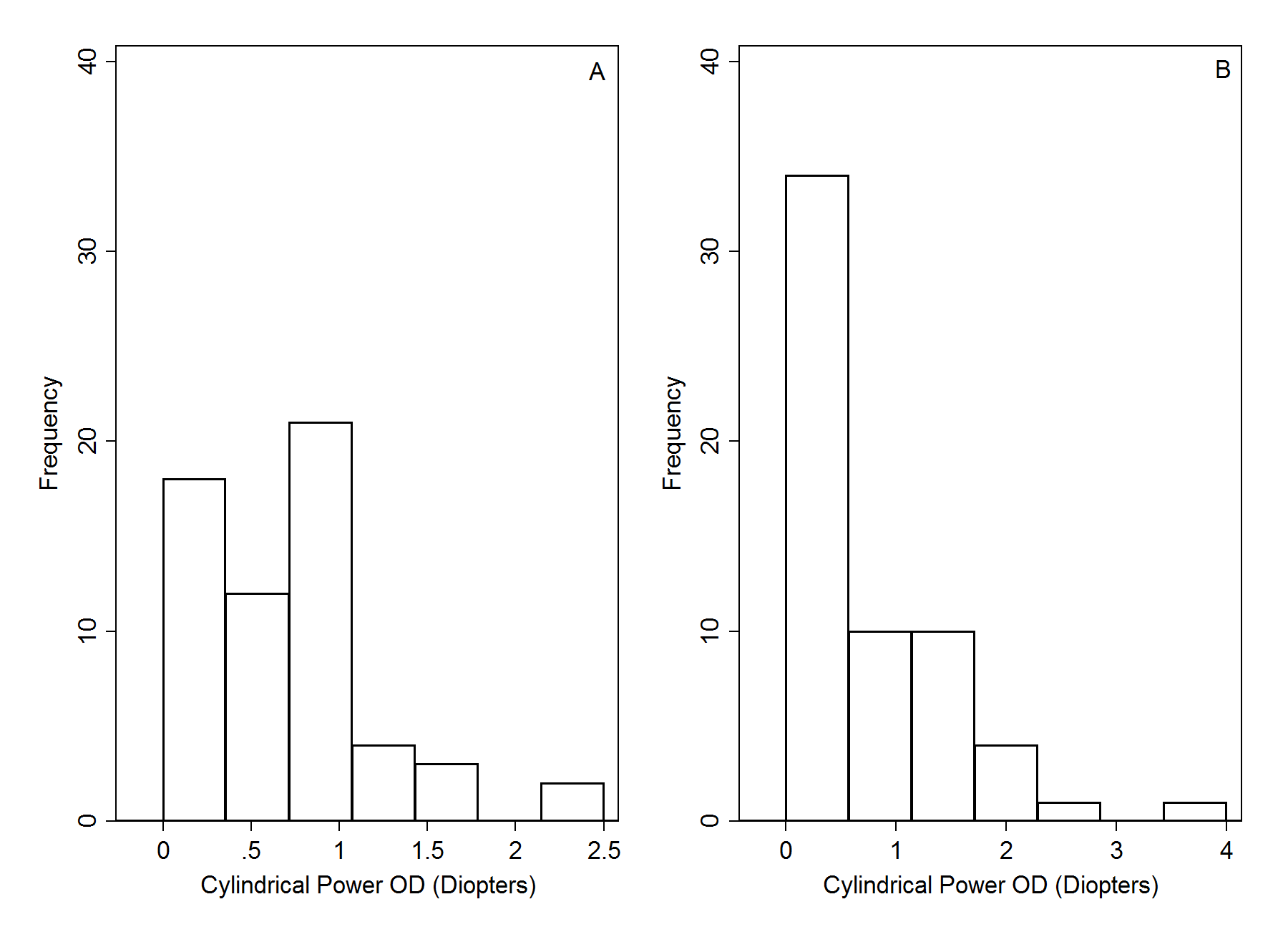

Supplement: S3 Fig — Abbreviations: OD = right eye, OS = left eye. (TIF) [file pone.0192055.s003.tif]
